# Correlations among Diabetic Microvascular Complications: A Systematic Review and Meta-analysis

**DOI:** 10.1038/s41598-019-40049-z

**Published:** 2019-02-28

**Authors:** Jianqing Li, Yihong Cao, Weiming Liu, Qiuke Wang, Yifeng Qian, Peirong Lu

**Affiliations:** 1 Department of Ophthalmology, The First Affiliated Hospital of Soochow University, 188 Shizi Street, Suzhou, 215006 P. R. China; 2 Department of Orthopedic Surgery, Shanghai Jiao Tong University Affiliated Sixth People’s Hospital, 600 Yishan Road, Shanghai, 200233 P. R. China

## Abstract

Early detection of diabetic microvascular complications is of great significance for disease prognosis. This systematic review and meta-analysis aimed to investigate the correlation among diabetic microvascular complications which may indicate the importance of screening for other complications in the presence of one disorder. PubMed, Embase, and the Cochrane Library were searched and a total of 26 cross-sectional studies met our inclusion criteria. Diabetic retinopathy (DR) had a proven risk association with diabetic kidney disease (DKD) [odds ratio (OR): 4.64, 95% confidence interval (CI): 2.47–8.75, p < 0.01], while DKD also related to DR (OR: 2.37, 95% CI: 1.79–3.15, p < 0.01). In addition, DR was associated with diabetic neuropathy (DN) (OR: 2.22, 95% CI: 1.70–2.90, p < 0.01), and DN was related to DR (OR: 1.73, 95% CI: 1.19–2.51, p < 0.01). However, the risk correlation between DKD and DN was not definite. Therefore, regular screening for the other two microvascular complications in the case of one complication makes sense, especially for patients with DR. The secondary results presented some physical conditions and comorbidities which were correlated with these three complications and thus should be paid more attention.

## Introduction

Diabetes mellitus (DM) is a chronic disease characterized by high blood sugar levels, and it is widely prevalent throughout the world. In 2017, about 451 million adults worldwide suffered from diabetes, and this number is estimated to increase to 693 million by 2045^1^. Notably, the microvascular and macrovascular complications of diabetes account for most of the morbidity and mortality associated with this disease^2^.

The diabetic microvascular complications caused by damage in the small blood vessels include diabetic retinopathy (DR), diabetic kidney disease (DKD), and diabetic neuropathy (DN). DR, whose overall prevalence worldwide is about one-third among diabetic patients^3^, is a leading cause of vision loss globally^4^. DKD, which is the chronic loss of kidney function, is the most common cause of end-stage kidney disease^5,6^ and it may require hemodialysis or even kidney transplantation^7^. DN is a nerve damaging disorder that may impair sensation, movement, gland or organ function, and other health aspects, depending on the nerve types affected.

The early detection of these complications is important, because it allows for early treatment and the prevention of disease progression. Several articles have pointed out the correlations among the diabetic microvascular complications^8–12^, which indicate the importance of screening the other two complications in the presence of one complication; however, there has not yet been a relevant meta-analysis. Therefore, we carried out a systematic review and meta-analysis in order to produce a pooled odds ratio (OR) of the interactions among the diabetic microvascular complications. In addition, the correlation factors of these microvascular complications, which should be controlled for disease prevention, were analyzed.

## Result

### Description of studies

**Figure 1:**
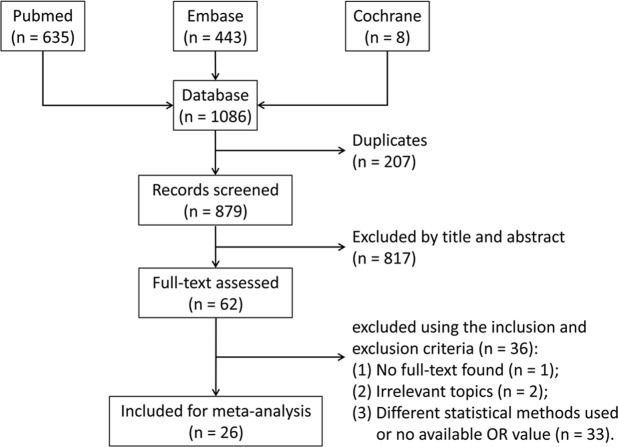
Flow diagram of the inclusion of studies in this meta-analysis.

A total of 1,086 articles were identified, and their records were included in Endnote X8 (Clarivate Analytics, Philadelphia, PA, US). After removing 207 duplicates, the remaining 879 articles were screened based on the titles and abstracts (separately) by two reviewers according to our inclusion criteria. Any disagreements about the inclusion of an article for full review were resolved by the third researcher. A full-text assessment was conducted on the rest of the 62 articles. Finally, 26 articles^13–38^ were included in this meta-analysis. The article search and selection process are summarized in Fig. 1.

The characteristics and JBI scores of these 26 studies are summarized in Table 1. A total of 60,136 participants were involved, and all of the articles included were of high quality according to their JBI scores. Besides, the methods of diagnosis DR, DKD and DN in the included studies were summarized in the Supplementary Table S1.

**Table 1:** Baseline characteristics and quality assessment of the included studies.

First Author (year)	Country	Year of Data collection	Study Setting	Sample Number	Male %	Age (year)^†^	Subtype of Diabetes	Duration of Diabetes (year)^†^	HbA1c %^†^	Adjusted Factors	JBI Scores
Schmid^13^	Brazil	1991	hospital-based	35	NA	57.5 ± 11.4	II	NA	8.3 ± 2.3	11, 25, 26, 27	7
Abu El-Asrar^14^	Saudi Arabia	NA	hospital-based	648	52.5%	48.8 ± 14.7	32.4% I 67.6% II	9.4 ± 6.5	NA	1, 2, 3, 4, 6, 11, 15, 17, 18, 27, 33, 37, 38, 39	7
Boelter^15^	Brazil	2002–2004	hospital-based	1214	43.3%	58.5 ± 10.3	II	11.1 ± 8.4	8.2 ± 2.0	2, 3, 5, 7, 12, 14, 15, 20, 27, 34	8
Al-Maskari^16^	United Arab Emirates	2003–2004	population-based	513	51.5%	53.3	14% I 86% II	≥10 years 79%	≥7% 62%	2, 3	8
Yokoyama^17^	Japan	2005	hospital-based	294	72%	59 ± 9	II	9 ± 8	6.6 ± 0.9	1, 2, 3, 7, 12, 13, 15, 16, 24, 32, 34, 37, 38, 42, 43, 44	8
Pradeepa^18^	India	NA	population-based	1629	44.6%	50.4 ± 11.3	II	4.6 ± 5.4	8.7 ± 2.2	NA	9
Jurado^19^	Spain	2003–2004	population-based	307	61.6%	59.6 ± 7.9	II	8.6 ± 7.0	7·0 ± 1·4	1, 3, 7, 11, 12, 13, 15, 20, 22, 23, 24, 27, 37, 39	8
Kärvestedt^20^	Sweden	NA	population-based	156	61%	61.7 ± 7.2	II	7.0 ± 5.7	6.4 ± 1.3	NA	7
Gong^21^	China	NA	population-based	668	40.1%	64.2 ± 11.5	II	7.3 ± 6.5	7.1 ± 1.6	1, 2, 3, 7, 8, 12, 13, 17, 18, 19, 20, 23, 28, 29	8
Pradeepa^22^	India	NA	population-based	1608	NA	NA	II	NA	NA	1, 2, 3, 7, 12, 18	8
Rodrigues^23^	Brazil	1998–2008	hospital-based	573	50.5%	33 ± 13	I	16 ± 19	9.0 ± 3.9	7, 17, 34	8
Voulgari^24^	Greece	NA	hospital-based	600	48%	50.7 ± 15.1	33.3% I 66.7% II	7.3 ± 9.3	7.8 ± 1.8	1, 2, 3, 5, 17, 19, 20	9
Azura^25^	Malaysia	2009–2010	hospital-based	254	42%	53.3 ± 9.1	II	NA	≥7% 81.5%	1, 21, 24	8
Ding^26^	Singapore	2004–2006	population-based	608	44.7%	62.8 ± 9.2	II	6.0 ± 2.5	7.9 ± 0.7	1, 2, 3, 7, 12, 14, 15, 17, 34, 39	9
He^27^	China	2008–2009	hospital-based	2009	57%	59.7 ± 12.3	II	8.1 ± 6.7	8.7 ± 0.8	1, 2, 3, 7, 10, 12, 13, 15, 16, 27, 33, 39, 41	8
Ji^28^	China	2010–2011	hospital-based	565	47.8%	66.6 ± 10.5	0.4% I 99.6% II	16.2 ± 5.9	8.2 ± 1.9	1, 7, 16, 24, 27, 34, 36	8
Karlberg^29^	Denmark	2007–2008	population-based	201	60.2%	33.1 (20.1–46.6)	I	(10–30)	8.7 ± 0.4	1, 2, 3, 7, 12, 13, 34	8
Sattaputh^30^	Thailand	2007–2008	hospital-based	899	28.6%	59.6 ± 9.9	II	8.1 ± 6.1	8.77 ± 1.85	1, 2, 3, 7, 12, 13, 24, 27, 30, 34	8
Won^31^	Korea	2009–2010	hospital-based	3999	48.5%	59 ± 10	II	9.6 ± 7.6	7.7 ± 2.7	1, 2, 3, 5, 7, 8, 11, 16, 22, 24, 27, 34, 37, 38, 39, 40	9
Xu^32^	China	2008–2009	population-based	1421	40.8%	61.3 ± 9.7	II	7.9 ± 6.3	7.05 ± 1.25	1, 11, 24, 27	7
Deng^33^	China	2011–2012	hospital-based	381	57.7%	60.8 ± 10.7	II	8.4 ± 5.9	8.0 ± 2.1	1, 2, 3, 34, 35, 36	8
Yang^34^	China	2013–2014	hospital-based	344	55.2%	57.1 ± 12.1	II	7.0 ± 4.1	9.0 ± 2.5	1, 12, 31	7
Al-Rubeaan^35^	Saudi Arabia	NA	population-based	50464	56.0%	59.7 ± 12.8	II	13.4 ± 8.2	8.9 ± 2.32	1, 2, 3, 5, 6, 11, 16, 21, 22, 27, 33, 34	8
Machingura^36^	Zimbabwe	2013–2014	hospital-based	344	27.3%	57.6 ± 14.8	24.4% I 75.6% II	10.3 ± 10.5	8.1 ± 1.0	3, 7, 9, 15, 24, 35, 45	8
Tentolouris^37^	Greece	NA	hospital-based	381	57.7%	64.1 ± 8.4	II	10.6 ± 10.0	7.2 ± 0.5	1, 2, 12, 14, 23, 27	8
Wei^38^	China	2009–2012	population-based	959	40.5%	64.6 ± 8.0	II	9.6 ± 7.1	6.9 ± 1.6	NA	8

^†^Data are mean ± standard deviation or mean or median (centile 10-centile 90).

JBI scores: article quality assessment using the Joanna Briggs Institute Prevalence Critical Appraisal Tool.

HbA1c: glycated hemoglobin.

Adjusted factors: 1 = age, 2 = gender, 3 = duration of diabetes, 4 = type of diabetes, 5 = treatment of diabetes, 6 = control of diabetes, 7 = glycosylated hemoglobin, 8 = fasting plasma glucose, 9 = fructosamine, 10 = C-peptide, 11 = hypertension, 12 = systolic blood pressure, 13 = diastolic blood pressure, 14 = antihypertensive medication, 15 = body mass index, 16 = dyslipidaemia, 17 = serum cholesterol level, 18 = serum triglycerides level, 19 = low density lipoprotein, 20 = high density lipoprotein, 21 = weight, 22 = obesity, 23 = waist circumference, 24 = diabetic retinopathy, 25 = non-proliferative diabetic retinopathy, 26 = proliferative diabetic retinopathy, 27 = diabetic kidney disease, 28 = blood urea nitrogen, 29 = uric acid, 30 = eGFR, 31 = Urinary albumin/creatinine ratio, 32 = albumin excretion rate, 33 = diabetic neuropathy, 34 = smoking, 35 = drinking, 36 = family history, 37 = coronary heart disease, 38 = cerebrovascular disease, 39 = peripheral vascular disease, 40 = foot ulcer, 41 = anemia, 42 = pulse-wave velocity, 43 = intima-media thickness, 44 = pulse pressure, 45 = HIV positivity.

### Critical appraisal tool

#### Primary clinical outcome

**Figure 2:**
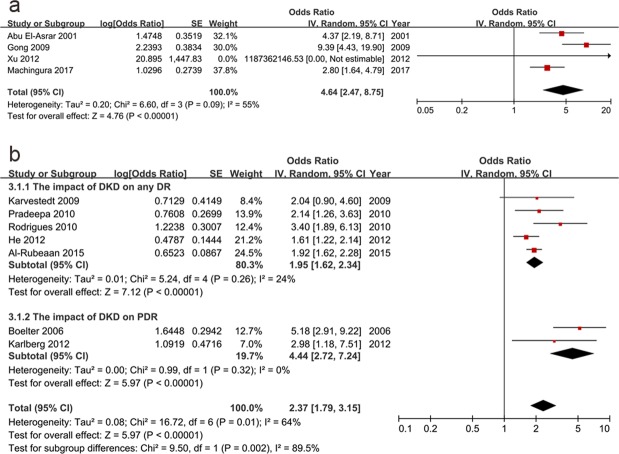
The association between diabetic retinopathy and diabetic kidney disease. DR had a risk impact on DKD (OR: 4.64, 95% CI: 2.47–8.75, p < 0.01) (**a**). In turn, DKD was correlated to DR (OR: 2.37, 95% CI: 1.79–3.15, p < 0.01). After stratifying DR into the any DR (OR: 1.95, 95% CI: 1.62–2.34, p < 0.01) and PDR (OR: 4.44, 95% CI: 2.72–7.24, p < 0.01) subgroups, the OR increased with the disease progression (p < 0.01) (**b**). (DR: diabetic retinopathy, DKD: diabetic kidney disease, PDR: proliferative diabetic retinopathy, OR: odds ratio, CI: confidence interval).

The main outcome of this study was the interactions among the diabetic microvascular complications, which were displayed by forest plots. The association between DR and DKD is shown in Fig. 2. DR was proven to be related to DKD (pooled OR: 4.64, 95% CI: 2.47–8.75, p < 0.01). In turn, DKD also correlated with DR (pooled OR: 2.37, 95% CI: 1.79–3.15, p < 0.01). As is shown in Fig. 2b, DR was stratified into any DR (OR: 1.95, 95% CI: 1.62–2.34, p < 0.01) and proliferative DR (PDR) (OR: 4.44, 95% CI: 2.72–7.24, p < 0.01) and the subgroup difference was of statistically significant (p = 0.002). When subgroup analysis was conducted based on the severity of DKD which included any DKD (OR: 2.52, 95% CI: 1.75–3.64, p < 0.01) and overt DKD (DKD with macroalbuminuria) (OR: 2.11, 95% CI: 1.35–3.29, p < 0.01), we found the subgroup difference was not statistically significant (p = 0.54) (see Supplementary Fig. S1). Besides, when subgroup analysis was based on different types of diabetes which included type 1 (OR: 3.27, 95% CI: 1.99–5.38, p < 0.01) and type 2 DM (OR: 2.20, 95% CI: 1.61–3.01, p < 0.01), the subgroup difference was neither statistically significant (p = 0.18) (see Supplementary Fig. S2).

**Figure 3:**
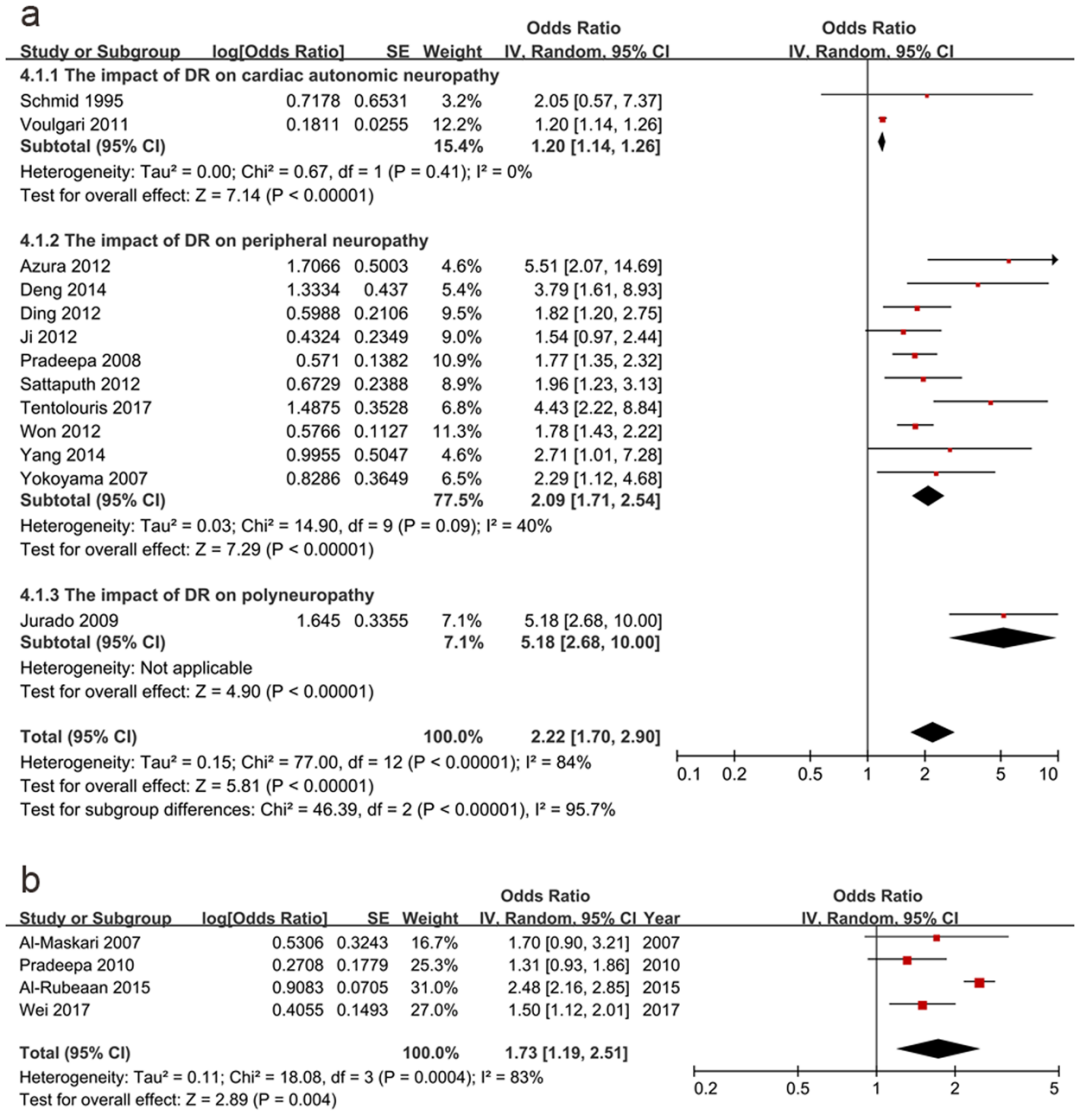
The relationship between diabetic retinopathy and diabetic neuropathy. DR was related to DN (OR: 2.22, 95% CI: 1.70–2.90, p < 0.01). Subgroup analysis was conducted based on different types of DN which contained cardiac autonomic neuropathy (OR: 1.20, 95% CI: 1.14–1.26, p < 0.01), peripheral neuropathy (OR: 2.09, 95% CI: 1.71–2.54, p < 0.01) and polyneuropathy (OR: 5.18, 95% CI: 2.68–10.00, p < 0.01), and the subgroup differences were of statistically significant (p < 0.01) (**a**). In turn, DN was related to DR (pooled OR: 1.73, 95% CI: 1.19–2.51, p < 0.01) (**b**). (DR: diabetic retinopathy, DN: diabetic neuropathy; OR: odds ratio, CI: confidence interval).

Next, the correlation between DR and DN was studied. DR was related to DN (pooled OR: 2.22, 95% CI: 1.70–2.90, p < 0.01) (Fig. 3a). Subgroup analysis was conducted based on different types of DN which contained cardiac autonomic neuropathy (OR: 1.20, 95% CI: 1.14–1.26, p < 0.01), peripheral neuropathy (OR: 2.09, 95% CI: 1.71–2.54, p < 0.01) and polyneuropathy (OR: 5.18, 95% CI: 2.68–10.00, p < 0.01), and the subgroup differences were of statistically significant (p < 0.01) (Fig. 3a). Besides, DR was then divided into the following subgroups: non-proliferative DR (NPDR) (OR: 1.30, 95% CI: 0.48–3.54, p = 0.60), PDR (OR: 3.98, 95% CI: 1.62–9.82, p < 0.01) and any DR (OR: 2.32, 95% CI: 1.70–3.17, p < 0.01). We found that NPDR did not have a definite association with DN because the 95% CI of the OR overlapped 1, and the p value was over 0.05. Nevertheless, the ORs for the any DR and PDR increased. However, the subgroup differences were not of statistically significance (p = 0.27) (see Supplementary Fig. S3 online). Furthermore, we conducted research on the impact of DR on DN in type 2 DM and any DM cases. We found that the OR in type 2 DM (OR: 2.40, 95% CI: 1.73–3.34, p < 0.01) and that in any DM (OR: 1.69, 95% CI: 1.24–2.30, p < 0.01) were not statistically different (p = 0.13) (see Supplementary Fig. S4). In turn, DN was related to DR (pooled OR: 1.73, 95% CI: 1.19–2.51, p < 0.01) (Fig. 3b).

**Figure 4:**
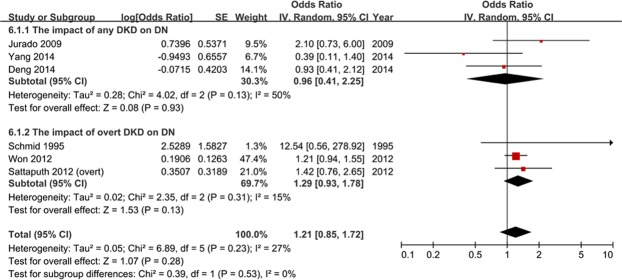
Diabetic kidney disease did not have a determined correlation with diabetic neuropathy (odds ratio: 1.19, 95% confidence interval: 0.95–1.49, p = 0.13).

The association between DKD and DN was also studied. After the pooled analysis, DKD did not have a determined relation with DN (pooled OR: 1.19, 95% CI: 0.95–1.49, p = 0.13) (Fig. 4). Stratification analysis was based on the severity of DKD which included any DKD and overt DKD, yet the subgroup difference was not statistically significant. Furthermore, there was only one article involving the influence of DN on DKD (OR: 1.44, 95% CI: 0.97–2.13, p = 0.07), yet the 95% CI of the OR overlapped 1, and the p value was over 0.05.

#### Secondary clinical outcome

The secondary outcome of this study was an analysis of the correlation factors of the diabetic microvascular complications, which was produced by a meta-analysis (Table 2). A total of 13 factors were studied with regard to their relationships with diabetic microvascular complications. For DR, the definite correlation factors were hypertension, diabetes duration, microalbuminuria, maleness, and age. However, the only certain correlation factor for DKD was hypertension. For DN, the determined correlation factors were the diabetes duration, HbA1C%, microalbuminuria, age, cardiovascular disease, peripheral vascular disease, and dyslipidemia.

**Table 2:** Associated physical conditions and comorbidities to diabetic microvascular complications.

Factors	Diseases	Number of Studies	OR (95% CI)	P value
Hypertension	DR	3	1.47 (1.29, 1.68)	<0.01
	DKD	2	1.83 (1.28, 2.64)	<0.01
	DN	5	1.37 (0.92, 2.05)	0.12
Diabetes Duration	DR	5	1.06 (1.02, 1.10)	<0.01
	DKD	2	1.02 (1.00, 1.04)	0.06
	DN	7	1.05 (1.03, 1.07)	<0.01
HbA1C%	DR	2	1.12 (0.81, 1.55)	0.51
	DKD	2	1.00 (0.95, 1.04)	0.86
	DN	7	1.21 (1.10, 1.33)	<0.01
Microalbuminuria	DR	2	2.54 (1.53, 4.23)	<0.01
	DN	3	1.26 (1.15, 1.38)	<0.01
Male	DR	3	1.57 (1.11, 2.23)	0.01
	DN	3	1.27 (0.67, 2.41)	0.46
Age	DR	2	1.03 (1.01, 1.05)	<0.01
	DN	7	1.10 (1.05, 1.15)	<0.01
Smoking	DR	2	0.84 (0.27, 2.65)	0.77
	DN	3	1.24 (0.98, 1.57)	0.07
CVD	DN	2	1.58 (1.16, 2.14)	<0.01
PVD	DN	3	3.87 (2.71, 5.52)	<0.01
HDL-C	DN	4	0.99 (0.97, 1.00)	0.11
Dyslipidemia	DN	4	1.32 (1.12, 1.55)	<0.01
PWV	DN	3	1.00 (1.00, 1.00)	0.59
BMI	DN	3	1.02 (0.99, 1.05)	0.23

^†^DR: diabetic retinopathy; DKD: diabetic kidney disease; DN: diabetic neuropathy; HbA1C: Glycated hemoglobin; CVD: cardiovascular diseases; PVD: peripheral vascular diseases; HDL-C: high density lipoprotein cholesterol; PWV: pulse wave velocity; BMI: body mass index.

### Sensitivity analysis

**Figure 5:**
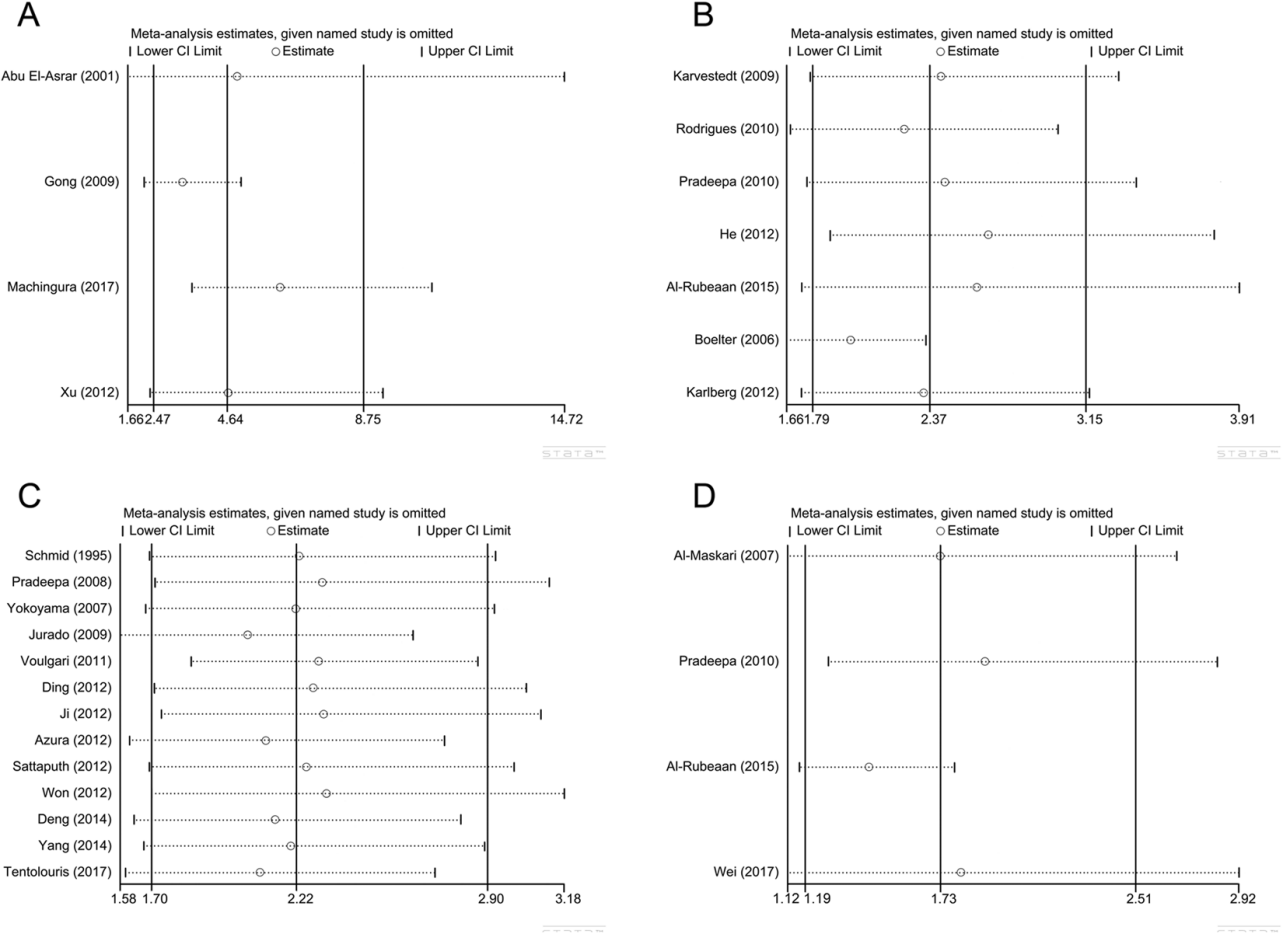
Sensitivity analysis on the outcome of the association between diabetic retinopathy and diabetic kidney disease and between diabetic retinopathy and diabetic neuropathy.

The heterogeneity was large in the outcome of the association between DR and DKD and between DR and DN (I^2^ ≥ 50) thus sensitivity analysis was conducted (Fig. 5). When omitting each study, we found no obvious changes to the results thus draw a conclusion that our results on vision efficacy were stable and reliable. Since the outcome of the impact of DKD on DN did not suffer apparent heterogeneity, we did not conduct sensitivity analysis on that.

### Publication bias

**Figure 6:**
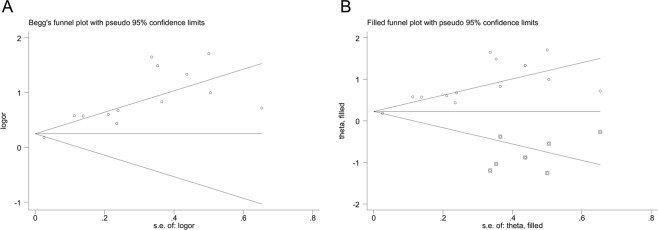
Begg’s funnel plots by Begg’s test (**A**) and filled funnel plots through trim and fill method (**B**) on the outcome of the influence of diabetic retinopathy on diabetic neuropathy.

Begg’s test and Egger’s test were appropriate for assessing publication bias when the included papers were larger than 10, thus they were only conducted on the outcome of the impact of DR on DN (n = 13). The asymmetry in the Begg’s funnel plot (Fig. 6A) and the p value of the Egger’s test (p < 0.05) indicated publication bias. Because of this, a sensitivity analysis using the trim and fill method was conducted (Fig. 6B) [%]. The pooled analysis incorporating the hypothetical studies continued to show a statistically significant influence of DN on DR by both fixed effect (OR: 1.25, 95% CI: 1.19–1.31) and random effect (OR: 1.40, 95% CI: 1.08–1.81), thus indicated the stability and robustness of our results.

## Discussion

DR and DKD were found to be correlation factors for each other. When studying DR’s impact on DKD, a total of 4 original data sets were involved, one of which possessed an abnormal OR value (1.187 × 10^9^, 95% CI: 0–∞). After consideration according to our inclusion criteria, it was involved in the meta-analysis. This resulted in 0% weight, and thus, it played no role in the final pooled value. In the analysis of the influence of DKD on DR, after dividing DR into the any DR and PDR subgroups, we found that along with DR progression, the correlation degree significantly increased (p < 0.01).

DR and DN are each other’s correlation factors. When studying the impact of DR on DN, we stratified DN into cardiac autonomic neuropathy, peripheral neuropathy and polyneuropathy and found statistically significant subgroup differences. Accordingly, cardiac autonomic neuropathy and peripheral neuropathy, being the two most common types of diabetic polyneuropathy, had diverse association with DR.

DKD is not a determined correlation factor for DN, and DN did not exert an impact on DKD. The 95% CIs and p values were close to the marginal values, and only one article involved the impact of DN on DKD. Therefore, more studies are needed, and their association still needs to be proven.

We also analyzed the correlation factors for these three complications. Among the DR’s correlation factors, hypertension and diabetes duration were in accordance with the results from another meta-analysis^39^, while microalbuminuria, maleness, and age were recognized for the first time in a meta-analysis. Moreover, HbA1C% is commonly considered to be related to DR^3,39,40^; yet, in this meta-analysis, their correlation was unclear (OR: 1.12, 95% CI: 0.81–1.55, p = 0.51). Since only two articles considered this data, more articles are needed, and the results might be different. Moreover, this is the first meta-analysis to identify the correlation factors of DKD and DN, with the exception of high-density lipoprotein cholesterol (HDL-C), which has been reported to decrease the risk of DN in type 1 diabetes^41^. In our study, HDL-C could be regarded to have protective influence on DN in view of its OR (0.99) and 95% CI (0.97–1.00), but the p value was 0.11, which was over 0.05. This is a controversial issue that requires further study, because several articles have reported its protective effect^42–45^ as well as its risk influence^46^ on DN.

Our study did have some limitations. First of all, potential publication bias was indicated by the asymmetry of Begg’s funnel plot on the outcome of DR’s influence on DN. The trim and fill sensitivity analysis did not change the general result that DR had a risk correlation with DN (although the strength was slightly attenuated), suggesting that those unpublished negative studies did not influence our results. Besides, we focused on cross-sectional studies which may only indicate the risk correlation but not the inferences of cause and effect. In addition, the disease diagnostic criteria varied across the studies, although they were all standardized and did not influence the stability of the outcomes. Fourthly, the adjusted confounding factors, which were the controlled covariates for multivariable logistic regression, varied in the included studies and thus might cause some heterogeneity. At last, the statistical method of our included studies was confined to multivariate logistic regression which might lead to some bias, however, we could obtain high quality data for meta-analysis.

By contrast, this was the first meta-analysis to evaluate the risk correlations among the diabetic microvascular complications. With data from 26 articles, a significant correlation was found between DR and DKD, as well as between DR and DN, which demonstrated that screening for the other two microvascular complications in the presence of DR is essential. In the future, more studies are needed to further analyze the association between DKD and DN. In addition, certain physical condition and comorbidities may relate to these three complications, and more attention should be paid to them.

## Methods

### Search strategy

Two independent reviewers (J. Li and Y. Cao) performed a systematic search of PubMed, Embase, and the Cochrane Library on January 9, 2018 for articles evaluating the associations among the diabetic microvascular complications. Using the MeSH or Emtree terms as well as free words, the search strategy included the following: [(“diabetic retinopathy” AND “diabetic nephropathy”) OR (“diabetic retinopathy” AND “diabetic neuropathy”) OR (“diabetic nephropathy” AND “diabetic neuropathy”)] AND “cross-sectional studies.”

### Selection criteria

The eligibility criteria were as follows: (1) cross-sectional studies, (2) multivariable logistic regressions were used to analyze the interactions among the diabetic microvascular complications, and (3) the ORs and 95% confidential intervals (CIs) could be obtained. Those studies with no full text, irrelevant topics, and different statistical methods were excluded. Any disagreements about the inclusion of an article for full review were resolved by a third researcher (P. Lu). The rigorous inclusion criteria were established and strictly followed by two independent reviewers (J. Li and Y. Cao) in order to control the selection bias.

### Quality assessment

In order to examine the validity of the included data for the meta-analysis, these articles were assessed using the Joanna Briggs Institute (JBI) Prevalence Critical Appraisal Tool, which contains 9 items^47^. The evaluation scores ranged from 0–9, with <3 defined as “low quality,” 3–6 as “moderate quality,” and >6 as “high quality.”

### Data extraction

The characteristics extracted from the eligible articles included the first author’s name, publication year, country where the study was conducted, year of data collection, study setting, number of samples, gender, mean age of the participants, diabetes subtypes, diabetes duration, glycated hemoglobin and the adjusted confounding factors.

The main outcome of this meta-analysis was the interactions among the diabetic microvascular complications. The secondary outcome was the correlation factors of these three diabetic complications. Therefore, the ORs and 95% CIs were extracted for further analysis.

### Data synthesis

The results from our included studies were combined using Review Manager (RevMan) version 5.3 (The Nordic Cochrane Centre, Copenhagen, Denmark). The effect value in this meta-analysis was the OR, which was obtained through a multivariate logistic regression. Before the analysis, the study heterogeneity was tested using both the I-squared and chi-squared test statistics. An I^2^ ≥ 50% and/or a Q-statistic of p < 0.10 were evidence supporting the presence of heterogeneity, in which the random effects modeling method was needed. Otherwise, the fixed effects modeling method was applied.

### Sensitivity analysis

Sensitivity analysis were employed to investigate the stability and reliability of the outcomes through Stata version 12.0 (StataCorp, Texas, America).

### Publication bias

Potential publication bias was evaluated using Stata by Begg’s test and Egger’s test. Additional trim and fill analysis was then conducted to test and adjust for publication bias.

## Supplementary information

Supplementary information files

## Data Availability

The datasets used and/or analyzed during the current study available from the corresponding author on reasonable request.
