# Supplementary material for: Correlations among Diabetic Microvascular Complications: A Systematic Review and Meta-analysis
Source: Sci Rep. 2019 Feb 28;9:3137. doi: 10.1038/s41598-019-40049-z (PMC6395813; doi:10.1038/s41598-019-40049-z)
Supplement: Supplementary file 1 — Supplementary information files [file 41598_2019_40049_MOESM1_ESM.doc]

**Correlations among Diabetic Microvascular Complications: A Systematic Review and Meta-analysis**

Jianqing Li^1,+^, Yihong Cao^1,+^ , Weiming Liu^1^, Qiuke Wang^2^, Yifeng Qian^1^, and Peirong Lu^1,*^.

^1^Department of Ophthalmology, the First Affiliated Hospital of Soochow University, 188 Shizi Street, Suzhou, 215006, P.R, China.

^2^Department of Orthopedic Surgery, Shanghai Jiao Tong University Affiliated Sixth People’s Hospital, 600 Yishan Road, Shanghai, 200233, P.R, China.

^*^Corresponding author: Tel/Fax: +86-0512-65215191

E-mail address: [lupeirong@suda.edu.cn](mailto:lupeirong@suda.edu.cn) (P. Lu)

^+^The first two authors contributed equally to this work.

**Figure S1.** The impact of DKD on DR was stratified according to the severity of DKD which included any DKD (OR: 2.52, 95%CI: 1.75–3.64, p < 0.01) and overt DKD (DKD with macroalbuminuria) (OR: 2.11, 95%CI: 1.35–3.29, p < 0.01), yet the subgroup difference was not statistically significant (p = 0.54). (DR: diabetic retinopathy, DKD: diabetic kidney disease, PDR: proliferative diabetic retinopathy, OR: odds ratio, CI: confidence interval).


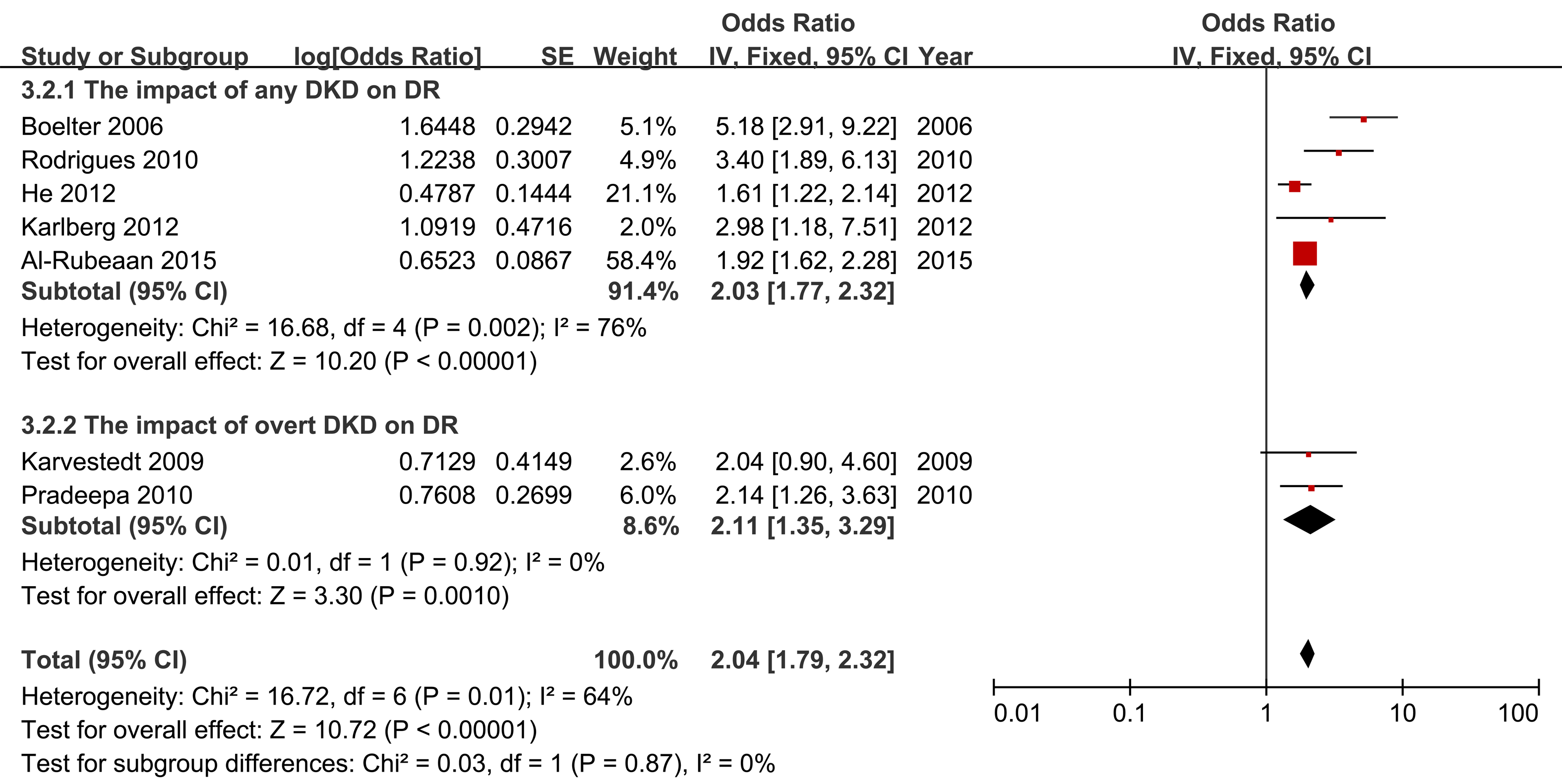


**Figure S2.** The influence of DKD on DR was stratified according to different types of diabetes which included type 1 (OR: 3.27, 95%CI: 1.99–5.38, p < 0.01) and type 2 DM (OR: 2.20, 95%CI: 1.61–3.01, p < 0.01), but the subgroup difference was not statistically significant (p = 0.18). (DR: diabetic retinopathy, DKD: diabetic kidney disease, PDR: proliferative diabetic retinopathy, DM: diabetes mellitus, OR: odds ratio, CI: confidence interval).


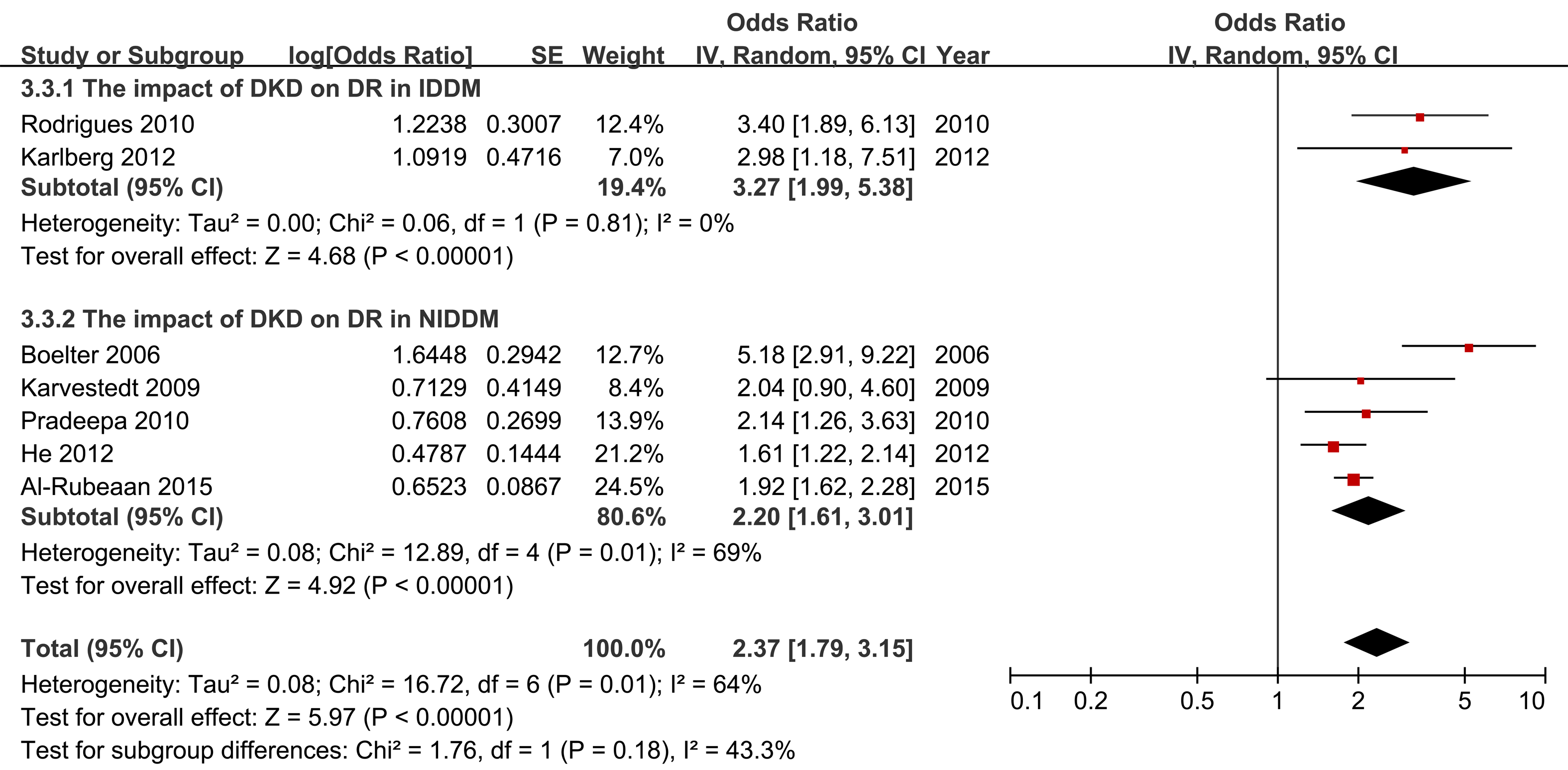


**Figure S3.** The impact of DR on DN was stratified according to the severity of DR which included NPDR (OR: 1.30, 95%CI: 0.48–3.54, p = 0.60), PDR (OR: 3.98, 95%CI: 1.62–9.82, p < 0.01) and any DR (OR: 2.32, 95%CI: 1.70–3.17, p < 0.01), yet the subgroup differences were not of statistically significance (p = 0.27). (DR: diabetic retinopathy, NPDR: non-proliferative diabetic retinopathy, PDR: proliferative diabetic retinopathy, DN: diabetic neuropathy, OR: odds ratio, CI: confidence interval)


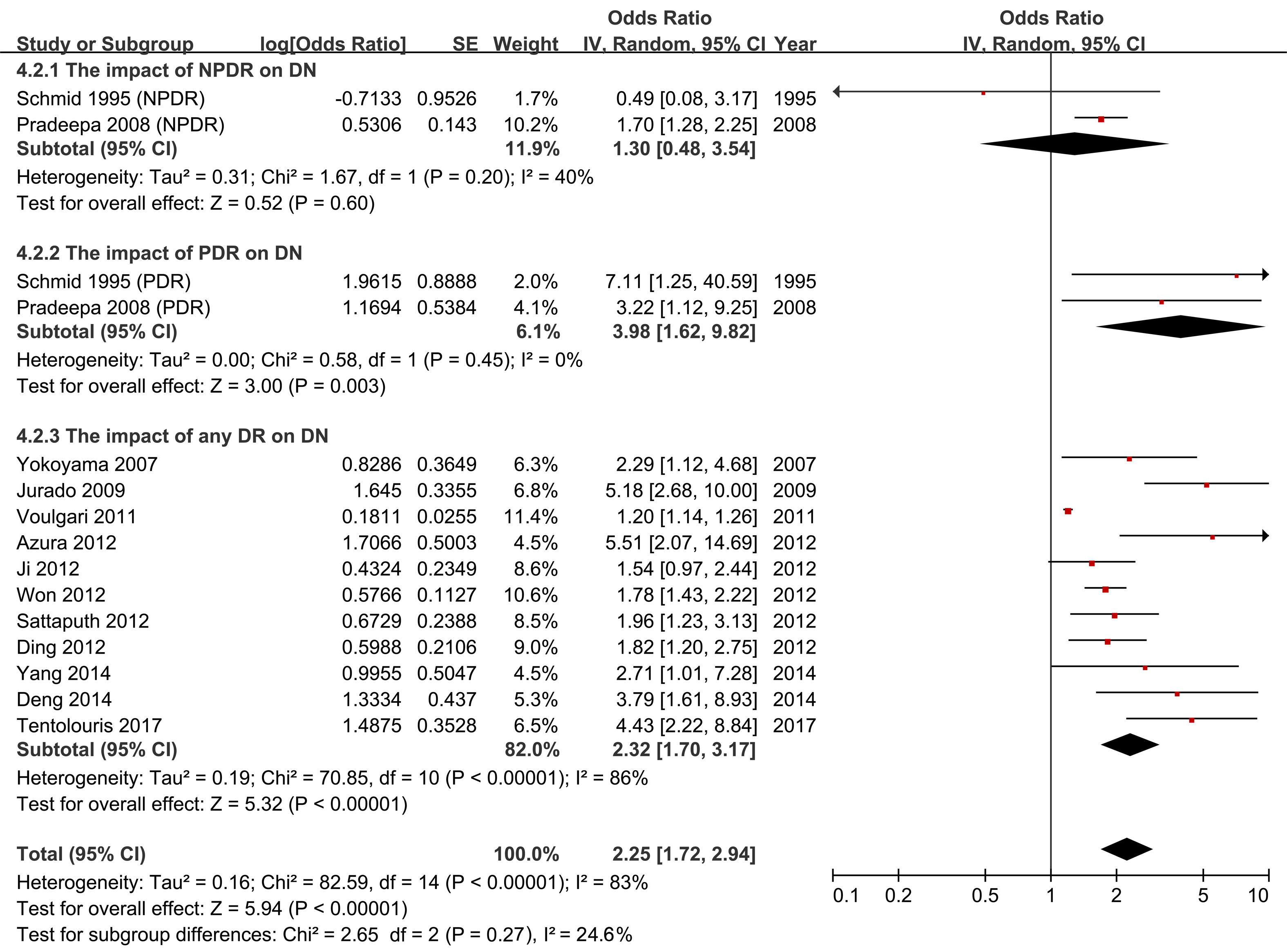


**Figure S4.** The influence of DR on DN was stratified according to different types of diabetes. The OR in type 2 DM (OR: 2.40, 95%CI: 1.73–3.34, p < 0.01) and that in any DM (OR: 1.69, 95%CI: 1.24–2.30, p < 0.01) were not statistically different (p = 0.13). (DR: diabetic retinopathy, DN: diabetic neuropathy, DM: diabetes mellitus, OR: odds ratio, CI: confidence interval)


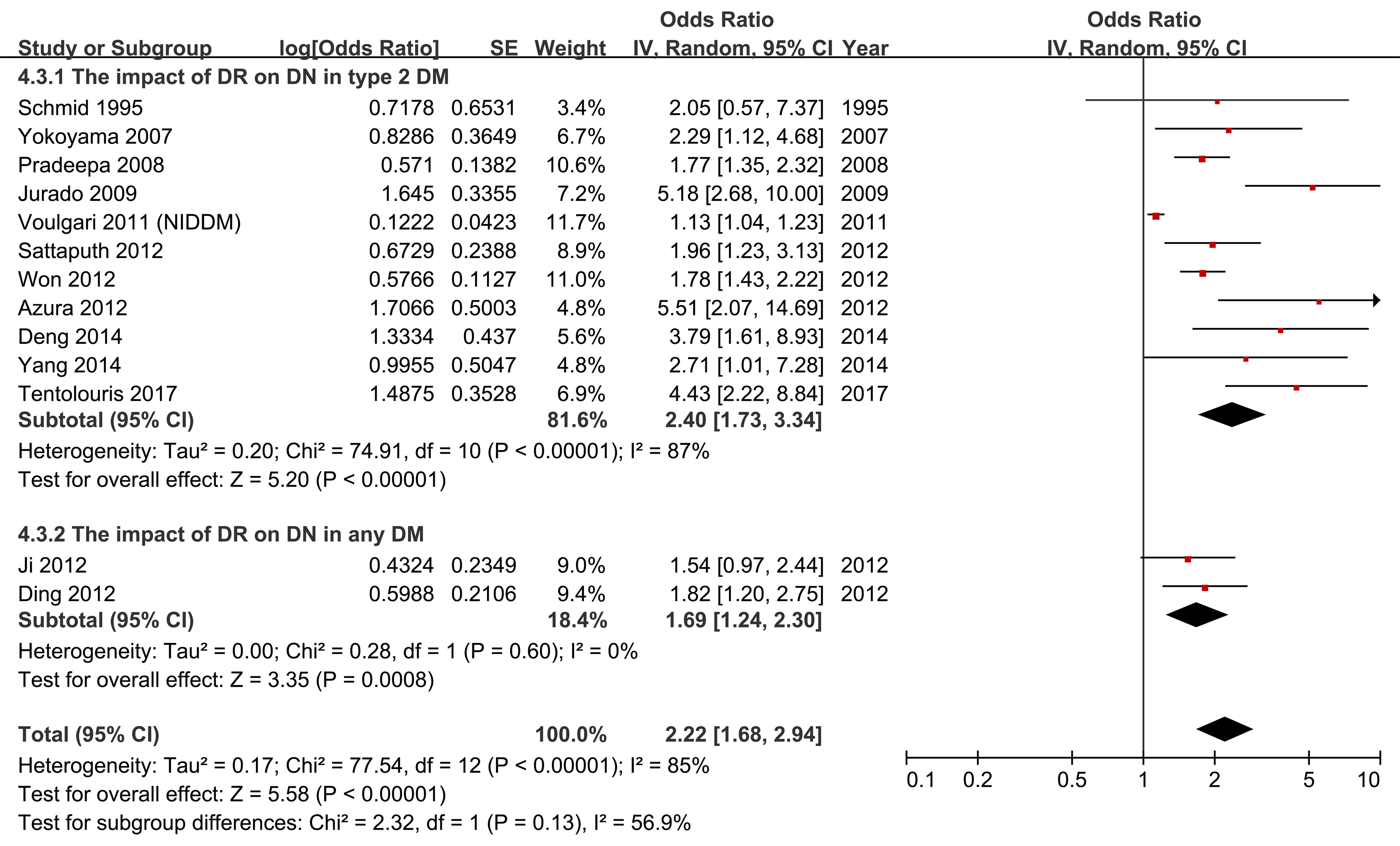


Table S1. The methods of diagnosing diabetic microvascular complications in the included studies.

| First Author (year) | Diagnosis of DR | Diagnosis of DKD | Diagnosis of DN |
| --- | --- | --- | --- |
| Schmid (1995)[13] | Indirect ophthalmoscopy was conduced according to the WHO multinational study of vascular disease in diabetes. | Urinary excretion of proteins > 550 mg/24 h or the presence of microalbuminuria (urinary albumin excretion rate > 20 μg/min). | At least two abnormal responses to cardiovascular autonomic function tests. |
| Abu El-Asrar (2001)[14] | Indirect ophthalmoscopy and contact lens were conducted by a retinal specialist. | NA | (1) Clinical symptoms of pain, burning, numbness or tingling, (2) impaired vibratory sensation and diminished temperature perception and fine touch sensation, (3) presence of foot ulcers. |
| Boelter (2006)[15] | Direct and indirect ophthalmoscopy were conducted by a trained ophthalmologist. | The presence of microalbuminuria (albumin = 17-176 mg/L in a random urine sample, or 30-299 mg in a 24h urine collection, or 24h urinary albumin excretion rate (UAER) = 20 to 199 μg/min) or macroalbuminuria (albumin ≥ 176 mg/L in a random urine sample, or ≥ 300 g in a 24h urine collection or 24h UAER ≥200 μg/min), or in patients on dialysis. | NA |
| Al-Maskari (2007)[16] | Fundus photography were assessed by two ophthalmologists. | NA | NA |
| Yokoyama (2007)[17] | Fundus photography. | The presence of creatinine ratio ≥ 30 mg/g. | At least two of the four components: the presence of symptoms, the absence of ankle tendon reflexes, and abnormal scores of vibration perception and heart rate variation. |
| Pradeepa (2008)[18] | Retinal photography were graded using the Early Treatment Diabetic Retinopathy Study grading system. | NA | The presence of mean vibratory perception threshold ≥ 20 V. |
| Jurado (2009)[19] | NA | NA | Two or more significant signs (vibratory perception threshold, Semmes-Weinstein Monofilament and Achilles reflex) or one sign plus two symptoms (sensitivity alterations, pain perception and decrease in muscle strength) |
| Karvestedt (2009)[20] | The photographic records of retinal photography and ophthalmoscopy were assessed by an experienced retina ophthalmologist. | The presence of albuminuria ≥ 300 mg/l or serum creatinine > 100 μmol/l for women and > 110 μmol/l for men. | The presence of vibratory perception threshold ≥ 25 V or inability to feel the monofilament. |
| Gong (2009)[21] | Fundus photographs were taken by a trained photographer and assessed by two endocrinologists and a retinal specialis. | The presence of macroalbuminuria (≥ 300 mg albumin/g creatinine) according to the screening protocol proposed by the American Diabetes Association. | NA |
| Pradeepa (2010)[22] | Fundus photographs were taken by trained and certified photographers and were graded by an ophthalmologist using the Early Treatment DR Study grading system. | The presence of albumin excretion ≥ 300 mg/g of creatinine. | The vibratory perception threshold of the big toe exceeded the mean +2 SD of the healthy study population without diabetes 20–45 years old (cutoff ≥ 20 V). |
| Rodrigues (2010)[23] | Direct and indirect ophthalmoscopy were conducted by an ophthalmologist, and the severity was classified using the Global Diabetic Retinopathy Group scale. | The presence of urinary albumin excretion ≥ 20 μg/min. | NA |
| Voulgari (2011)[24] | Direct fundoscopy were conducted by an experienced ophthalmologist. | The presence of estimated glomerular filtration rate < 60 ml/min per 1.73 m^2^ or proteinuria. | Assessment for peripheral neuropathy was based on symptoms (neuropathy symptom score) and signs (neuropathy disability score).  Cardiac autonomic neuropathy was diagnosed when two of the four classical autonomic function tests were abnormal. |
| Azura (2012)[25] | Fundus pictures. | The presence of estimated glomerular filtration rate < 60 ml/min per 1.73 m^2^. | Four plantar sites were tested to diagnosed peripheral neuropathy. |
| Ding (2012)[26] | Retinal photographs were evaluated by trained graders according to standardized protocols. | NA | Abnormal vibration sensation (vibratory perception threshold > 25 V at any site) or insensitivity to the 10 g monofilament (the score of monofilament sensory test of < 4 of 5 points on either foot). |
| He (2012)[27] | Fundus photographic imaging was carried out following a standardized protocol and evaluated according to the International Clinical Diabetic Retinopathy and Diabetic Macular Edema Disease Severity Scales. | The presence of 24 h urinary albumin excretion > 30 mg. | Electromyogram. |
| Ji (2012)[28] | A specialist physician. | The diagnoses existed in the medical history and/or actual treatment for the disorder was performed, and/or urinary albumin excretion ≥ 20 μg/ml. | Established diagnosis in the medical history and/or the presence of overt neuropathic symptoms, and/or neurologic examinations according to the standards of medical care in diabetes—2008 published by the American Diabetes Association. |
| Karlberg (2012)[29] | Ophthalmoscopy was conducted by a trained retinal specialist. | The presence of urinary albumin excretion ≥ 20 mg/l. | NA |
| Sattaputh (2012)[30] | NA | The presence of urine albumin to creatinine ratio ≥ 30 mg/g. | Detected by testing for loss of protective sensation as the inability to feel the 10 gram Semmes-Weinstein 5.07 monofilament at one or more locations on the foot except the heel. |
| Won (2012)[31] | A review of medical records. | The presence of albuminuria ≥ 300 mg⁄ g or serum creatinine ≥ 100 μmol ⁄ l. | Established diagnosis or neuropathy symptoms (Michigan Neuropathy Score Instrument ≥ 3) and an abnormal result on the monofilament test. |
| Xu (2012)[32] | Fundus examination were judged by ophthalmologists. | The presence of overt albuminuria or microalbuminuria according to the Kidney Disease Outcomes Quality Initiative. | Diagnosed by the sense of distal numbness and pain. |
| Deng (2014)[33] | NA | NA | Based on the diagnostic criteria recommended by theAmerican Diabetes Association in 2010. |
| Yang (2014)[34] | Fundus photographs and/or fundus fluorescein angiography were assessed according to the protocol made in the 2002 Sydney International Eye Conference. | The presence of urine albumin to creatinine ratio ≥ 30mg/g. | Diagnosed according to the Neuropathy Symptom Score and the Neuropathy Disability Score. |
| Al-Rubeaan (2015)[35] | Direct ophthalmoscopy was assessed by ophthalmologists. | NA | NA |
| Machingura (2017)[36] | An ophthalmologist conducted examination for retinopathy using a slit-lamp indirect ophthalmoscopy with a diopter lens. | The presence of moderately increased albuminuria and severely increased albuminuria. | NA |
| Tentolouris (2017)[37] | Direct fundoscopy was conducted by an experienced ophthalmologist. | The presence of estimated glomerular filtration rate < 60 ml/min per 1.73 m^2^ or microalbuminuria or proteinuria. | Assessment included examination of neuropathic symptoms and signs using the Neuropathy Symptom Score and the Neuropathy Disability Score. |
| Wei (2017)[38] | Fundus photographs were graded by the Early Treatment DR Study grading system. | NA | At least one abnormity tested by  10 g Semmes-Weinstein monofilament, 128 Hz tuning fork and Tip-Therm. |
